# Representation of skin tone: The use of medical imagery in the genetic counseling profession

**DOI:** 10.1002/jgc4.70155

**Published:** 2025-12-07

**Authors:** Lawri Sanders, Karen Mary Davalos, Iman Kashmola‐Perez, Ian M. MacFarlane, Heewon Lee

**Affiliations:** ^1^ Department of Genetics, Cell Biology, and Development University of Minnesota Minneapolis Minnesota USA; ^2^ Department of Chicano and Latinx Studies University of Minnesota Minneapolis Minnesota USA; ^3^ Variantyx, Inc Framingham Massachusetts USA

**Keywords:** DEIJ, diverse imagery, education, genetic counseling, medical imagery, race, racism, textbook

## Abstract

The utilization of medical imagery featuring human bodies is a common practice in educational settings and patient interactions. However, these images predominantly depict white bodies and lighter skin tones, raising questions about their inclusivity and representation. This cross‐sectional quantitative study addressed this gap by assessing the diversity of medical imagery in the genetic counseling field. Participants (*n* = 103) completed a 43‐item survey where they responded to prompts about their experiences with medical imagery, depicting Black, Indigenous, People of Color (BIPOC) bodies and white bodies within their genetic counseling (GC) role, their genetic counseling program instructor role, and during their time as genetic counseling students. In their GC role, participants were significantly more likely to see (*p* < 0.001) and use (*p* = 0.02) white imagery compared to BIPOC imagery. In both their GC and instructor roles, participants found it more difficult to find BIPOC imagery (*p* < 0.001) and had to put more effort into finding it (*p*_GC_ < 0.001; *p*_instructor_ = 0.001). As students, participants were more likely to have seen white imagery in their curriculum (*p* < 0.001). When looking for diverse imagery, participants often resorted to Google searches (*n* = 44) and used search terms that encompass both the medical condition and the desired race (*n* = 16). The most common barrier participants encountered when looking for diverse imagery was the general lack of diversity in stock photo resources (*n* = 61). This study sheds light on the lack of diversity in medical imagery within the genetic counseling field and emphasizes the urgent need for inclusive representation. By enhancing providers' knowledge of how conditions manifest across diverse racial and ethnic groups, diverse medical imagery can contribute to mitigating health inequalities among patients of color. Bringing attention to the resources used to educate others on human health is critical for the future development of inclusive resources, such as medical imagery.

**What is known about this topic**

The diversity of medical imagery has primarily been studied within dermatology, a field in which skin tone plays a significant role in the accuracy of a diagnosis. In medical specialties where imagery does not adequately represent a variety of populations, practitioners may feel less confident when diagnosing BIPOC patients. Thus, this lack of diversity may contribute to disparities in the care BIPOC patients receive compared to their white counterparts.

**What this paper adds to the topic**

This is one of the first known studies to examine the diversity of medical imagery within genetic counseling. Understanding how medical imagery is used in genetic counseling encounters and student curriculum can help professionals and students in the field better tailor their care for underrepresented patient populations.

## 1 INTRODUCTION

Most medical imagery is comprised of white, cisgender, heterosexual, able‐bodied, muscular, young men (Chichester et al., 2023; Louie & Wilkes, 2018; Parker et al., 2017; Ray King et al., 2021). Only 4.5% of imagery in medical textbooks is estimated to depict darker skin tones (Louie & Wilkes, 2018). In a photogrammetric study that examined medical imagery of humans published in the *New England Journal of Medicine* from 1992 to 2017, approximately 18% depicted “non‐white” skin tones (Massie et al., 2021). Notably, Africa was the only geographical region in this study where the majority (67%) of articles contained such imagery. Additionally, about 37% of articles within the genetics specialty included diverse imagery.

The lack of diversity in medical illustrations has an impact on the quality of healthcare for Black, Indigenous, People of Color (BIPOC) patients because a provider's point of reference for a condition is likely based on white patients. Some conditions can present differently across racial and ethnic groups, which can affect the rate, accuracy, and confidence of a diagnosis (Buonsenso et al., 2022; Ilic et al., 2022; Koretzky et al., 2016; Kurd & Gelfand, 2009). A study conducted with medical students found they felt less confident in diagnosing skin conditions in BIPOC patients, even if their diagnoses turned out to be accurate (Bellicoso et al., 2021). Accuracy in a diagnosis does seem to increase, however, when shown an array of skin tones (Ilic et al., 2022). Deficits in awareness of how conditions manifest in people of color ultimately trace back to an education system rooted in racism and a history of the pathologization of race in medicine. In the 18th century, the theory that different racial groups were of different human origins rose to prominence (Nieblas‐Bedolla et al., 2020). The pathologization of race persists today and thus contributes to how BIPOC patients are treated medically and socially. A case study conducted on BIPOC patients with genetic conditions included a 2‐year‐old African American patient with Down Syndrome who had a delayed diagnosis despite presenting with developmental delay and dysmorphologic features (Omorodion et al., 2022). Patients with Down Syndrome who are of African descent are less likely to exhibit some of the condition's classic physical features (Kruszka et al., 2017). Additionally, features such as a flat nasal bridge and epicanthal folds—commonly present in unaffected individuals of African ancestry—can further obscure clinical recognition and contribute to a delayed diagnosis. Another African American patient in this case study presented with progressive skin darkening. This was dismissed by the pediatrician. Hyperpigmentation, or “skin bronzing” as stated in medical education, can be an indicator of adrenal insufficiency. The patient's symptoms were not recognized as clinically significant until they experienced a seizure. Only then was genetic testing pursued, leading to a diagnosis of X‐linked adrenoleukodystrophy. Lastly, a case report found that syndromic capillary malformations can be misdiagnosed as café au lait macules in BIPOC individuals, which results in an unnecessary evaluation of neurofibromatosis and other related conditions (Nriagu et al., 2021). These cases exemplify how BIPOC patients are less likely to receive an accurate diagnosis until much later or until the condition has reached a severe state due to biases and/or insufficient training by healthcare providers, emphasizing a need for diverse medical imagery (Ilic et al., 2022; Koretzky et al., 2016; Kurd & Gelfand, 2009; Omorodion et al., 2022).

In November 2021, Chidiebere Ibe, a medical student from Nigeria, created an illustration of a dark‐skinned fetus in the womb and posted it on Instagram (Ibe, 2021). Users across social media noted how his drawing was the first time they had seen a medical illustration of a Black body. Recently, Ibe and others have made efforts to diversify medical illustrations in textbooks, image repositories, creations by artists, and more (Biotic Artlab, n.d.; Diverse Health Hub, n.d.; Illustrate Change, n.d.; Ibe, 2023; Jackson‐Richards & Pandya, 2014; Taylor et al., 2016). Diverse images can also be found in stock images, but they exist in a small quantity behind a paywall and leave out certain groups (Chichester et al., 2023).

Medical imagery and other forms of visual aids are frequently used by genetic counselors (GCs) to facilitate patient understanding in various genetic counseling topics and to educate GC students on the mechanisms and natural history of genetic conditions. Because visual aids are often used to explain more abstract concepts in genetics, such as genes, chromosomes, inheritance patterns, etc., it is unknown how often GCs use visual aids of human bodies. Consequently, the use of diverse medical illustrations within the field is not well understood. With genetic counseling being a sector of healthcare that places an emphasis on patient‐centered care, having resources that showcase and include a part of their identities could be another avenue to build trust with patients. It is also important for GCs and GC students to be knowledgeable about what genetic conditions could look like in each patient demographic profile so as not to further contribute to health disparities. This paper aims to investigate how medical illustrations and visual aids are used in the genetic counseling profession and the curriculum of genetic counseling programs. Through this analysis, we hope to determine factors that contribute to the possible lack of diversity of imagery in the field and brainstorm methods to mitigate them. There are countless aspects to diversity, and future research is needed to account for the gaps in other identities. That said, this research focuses on race, ethnicity, and skin tone. While skin tone does not always align with racial identity, this paper uses it as a proxy for race to reflect how existing literature categorizes people of color in medical imagery.

## 2 METHODS

This study obtained approval from the University of Minnesota Institutional Review Board in August 2023 (STUDY00020057).

### 2.1 Study population

Participants were eligible to participate in the study if they were ABGC board‐eligible or board‐certified genetic counselors and/or had taught at least one lecture for a class in an American or Canadian genetic counseling program within the 2022–2023 academic year. In addition, participants had to be at least 18 years of age at the time of completing the study. To recruit these participants, an invitation was sent to members of the Genetic Counseling Education Association (GCEA; *N*~150 members, formally known as the Association of Genetic Counseling Program Directors (AGCPD)) and the National Society of Genetic Counselors (NSGC, 2023; *N*~4500) through the Student Research Survey Program on November 8th, 2023. The invitation included a brief description of the study as well as a link to the survey. Additional recruitment efforts included social media (Discord, X, and LinkedIn), snowball sampling, and word of mouth. Based on a priori power analysis calibrated to detect a moderate effect ($\eta_{p}^{2}$ = 0.06; Cohen, 1988) with 0.80 power and α = 0.006, our target sample size was 140. Recruitment and data collection took place from November 8th to December 20th, 2023.

### 2.2 Instrumentation

An electronic survey was created through Qualtrics. Prior to the distribution of the survey, it was tested by two instructors and three graduate students in the University of Minnesota genetic counseling program. Because the survey was developed entirely from scratch, they provided feedback on content, phrasing, and flow.

The survey started with a consent form and an eligibility check. Those who met the eligibility criteria were asked to complete CAPTCHA screening to prevent bot entries. Participants were asked about their current role(s) (e.g., practicing GC, GC program faculty, guest lecturer for a GC program) as well as professional demographics. Those who graduated from GC programs were asked for their graduation year. Those in direct patient care or a mixed position were asked about patient loads and session logistics, such as the number of patients they see per week and the average length of their sessions.

Within each role a participant selected, they were asked to indicate (using a 4‐point Likert scale) how often they saw and used white and BIPOC images, how difficult it was to find these images, and how much effort was put into finding these images, along with a few items tailored to that specific role. Participants were told that examples of imagery include illustrations of human bodies, diagrams of body parts/organ systems where skin color is visible, and photos of human bodies with/without a genetic condition. These could come from literature, textbooks, videos, patient brochures, websites, visual aid repositories, or other sources. Participants who had graduated from a GC program were asked about their experiences with diverse imagery during their time as a student, again using 4‐point Likert scales. Participants were also asked how diverse imagery would impact their current practices.

If participants indicated they use medical imagery in any of their current roles, they were asked about the resources used to find diverse images and the search terms they use. If participants indicated they do not use medical imagery, they were asked about the resources they would use to find diverse images and the search terms they would use. Participants next selected the barriers they have experienced when looking for diverse imagery. They were also asked to rank order nine potential sources of diverse imagery from the most to least preferred. Finally, participants were asked questions about demographic variables.

Upon completion, participants were redirected to another survey to maintain the anonymity of their previous responses. In this survey, they were asked if they would like to enter a drawing for one of ten $20 Greenphire ClinCards and be contacted for future studies. If they responded yes to at least one of these questions, they were asked to provide their email. In total, the survey was expected to take about 10–15 min to complete.

### 2.3 Data analysis

Before conducting analysis, we checked for range violations, potential outliers, and determined if there were patterns of missing data that would influence analysis or interpretation. We conducted descriptive analyses of all study variables to assess the use of medical imagery and whether diverse medical imagery is readily available. We used mixed and repeated measures MANOVA to evaluate group‐level differences in experiences with medical imagery. We also used paired *t*‐tests to assess the use of BIPOC versus white imagery in different aspects of participants' training experience. Each of the four MANOVAs and the four *t*‐tests was assessed at a Bonferroni‐corrected α = 0.006. Post‐hoc analyses were considered exploratory and assessed at an unadjusted α = 0.05.

## 3 RESULTS

One hundred fifty‐four responses were recorded on Qualtrics (estimated response rate of 3.4%), and 103 were used for analysis. Twenty were removed due to having more than 20% missing data (following the recommendations of Peng et al., 2006), eight for finishing in an unreasonably short amount of time (<200 s), five for having the same IP address as other responses, nine for duplicate or illogical responses, and nine for not meeting the inclusion criteria. Because we did not reach the sample size of 140 determined by the a priori analysis, we conducted sensitivity analyses to assess the effect size we could detect with 0.80 power for each MANOVA we report below. All analyses would detect effects at or below the convention for a moderate effect size (observed $\eta_{p}^{2}$: 0.04–0.06; moderate $\eta_{p}^{2}$ = 0.06; Cohen, 1988), suggesting reasonable confidence in the results for effects likely to make a substantive difference.

### 3.1 Participant demographics

Participants were about 33 years old on average (range: 24–58) and primarily white non‐Hispanic (*n* = 80; 77.7%), cisgender women (*n* = 66; 64.1%; for full sample demographics, please see Table 1). In comparison to the 2023 NSGC Professional Status Survey (PSS), our sample had greater diversity in terms of race and gender. Ninety‐two participants attended and graduated from a genetic counseling program (89.3%) with an average graduation year of 2016 (range: 1984–2023). Nearly all participants lived in the United States (*n* = 100; 96.4%). Most lived in a city where at least 50% of residents are white (*n* = 66; 71.7%) and had a patient or client population that was at least 50% white (*n* = 63; 64.9%). Forty‐seven participants worked in an urban area (45.6%), 17 in a suburban area (16.5%), two in a rural area (1.9%), and 37 in a mixture of these (35.9%).

**TABLE 1:** Demographic variables for the sample of genetic counselors (*N* = 103).

Variable	*n*	%	Variable	*n*	%
Personal variables					
*Sex assigned at birth*			*Race* ^a^		
Female	68	66.0	White/European	85	82.5
Male	29	28.2	Black/African/African American	8	7.8
Prefer not to answer	6	5.8	Middle Eastern or North African	1	1.0
*Gender*			Asian	8	7.8
Cisgender woman	66	64.1	Hispanic/Latine	2	1.0
Cisgender man	24	23.3	American Indian/Alaskan Native/Indigenous Peoples of Canada	1	1.0
Transgender (FTM)	1	1.0	Native Hawaiian/Pacific Islander	1	1.0
Transgender (MTF)	1	1.0			
Nonbinary	1	1.0			
Prefer not to answer	10	9.7			
Professional variables					
*GC program attendance*			*Primary area of practice*		
Attended and graduated	92	89.3	Prenatal	12	11.7
Did not attend or graduate	11	10.7	Oncology	20	19.4
*Genetic counselor role* ^a^			Adult genetics	16	15.5
Board‐eligible GC	3	2.9	Pediatric genetics	28	27.2
Currently practicing GC	79	76.7	Lab	10	9.7
Not currently practicing GC	13	12.6	Education	13	12.6
Guest lecturer for a GC program	26	25.2	Other^b^	4	3.9
Instructor for a GC program	22	21.4	*Working hours*		
Faculty for a GC program	22	21.4	Full‐time	99	96.1
Program leadership position	14	13.6	Part‐time	4	3.9
Position variables					
*Type of position*			*% of time spent in patient care*		
Direct patient care	52	50.5	<25%	7	8.2
Non‐direct patient care	10	9.7	25%–50%	32	37.6
Mixed position	33	32.0	51%–75%	23	27.1
Non‐patient care	8	7.8	>75%	23	27.1
*Average # of patients per week*			*Average length of session*		
<5	12	14.1	<15 min	6	7.1
5–10	27	31.8	15–30 min	20	23.5
11–15	27	31.8	31–45 min	37	43.5
16–20	16	18.8	46–60 min	16	18.8
21–25	2	2.4	>60 min	6	7.1
<25	1	1.2			
Instructor variables					
*Course(s) taught*					
Ethics	5	6.5			
Laboratory	11	14.3			
Medical genetics	38	49.4			
Professional development	13	16.9			
Psychosocial counseling	17	22.1			
Skills and practice	23	29.9			
Other^c^	12	15.6			
Workplace setting variables					
*Geographic region* ^d^			*Population density*		
Northeast US	9	10.7	Urban	47	45.6
Midwest US	31	36.9	Suburban	17	16.5
South US	19	19.6	Rural	2	1.9
West US	22	26.2	Mix	37	35.9
Canada	3	3.6			
*City population*			*Patient population*		
0%–25% White	1	1.1	0%–25% White	5	5.2
26%–50% White	25	27.2	26%–50% White	29	29.9
51%–75% White	51	55.4	51%–75% White	43	44.3
76%–100% White	15	16.3	76%–100% White	20	20.6

Quantitative variables	*M*	SD	Mdn	Range
Age	32.85	7.34	31	24–58
Graduation year	2016	7.87	2018	1984–2023
Years in current role	4.54	4.51	3	0–28

^a^ Participants could select multiple.

^b^ Other includes research (*n* = 2), biochemical genetics (*n* = 1), and a split between medical genetics and laboratory genomics (*n* = 1).

^c^ Other includes cancer genetics (*n* = 5), research (*n* = 3), prenatal/embryology (*n* = 3), and general genetics (*n* = 1).

^d^ Participants filled in the blank and were categorized per the US Census.

All participants noted they were genetic counselors; 79 were currently practicing in a clinical setting (76.7%). The most common primary specialties were pediatric genetics (*n* = 28; 27.2%), oncology (*n* = 20; 19.4%), and adult genetics (*n* = 16; 15.5%). This differs from the PSS, where oncology was the most common specialty (*n* = 915; 34%). The majority worked full‐time (*n* = 99; 96.1%) and have been in their current role for about 5 years (range: 0–28). Half worked directly with patients (*n* = 52; 50.5%), 18 did not directly work with patients (17.5%), and 33 worked in a mixed position (32.0%). Most participants spent at least half their time in direct patient care (*n* = 46; 54.2%) with an average session length of 31–45 min (*n* = 37; 43.5%) and patient load of 5–15 per week (*n* = 54; 63.6%). Seventy‐seven participants have taught for a GC program (74.8%), with the most common classes being medical genetics (*n* = 38; 49.4%), skills and practice (*n* = 23; 29.9%), and psychosocial counseling (*n* = 17; 22.1%).

### 3.2 Genetic counselors' experiences with medical imagery

**FIGURE 1:**
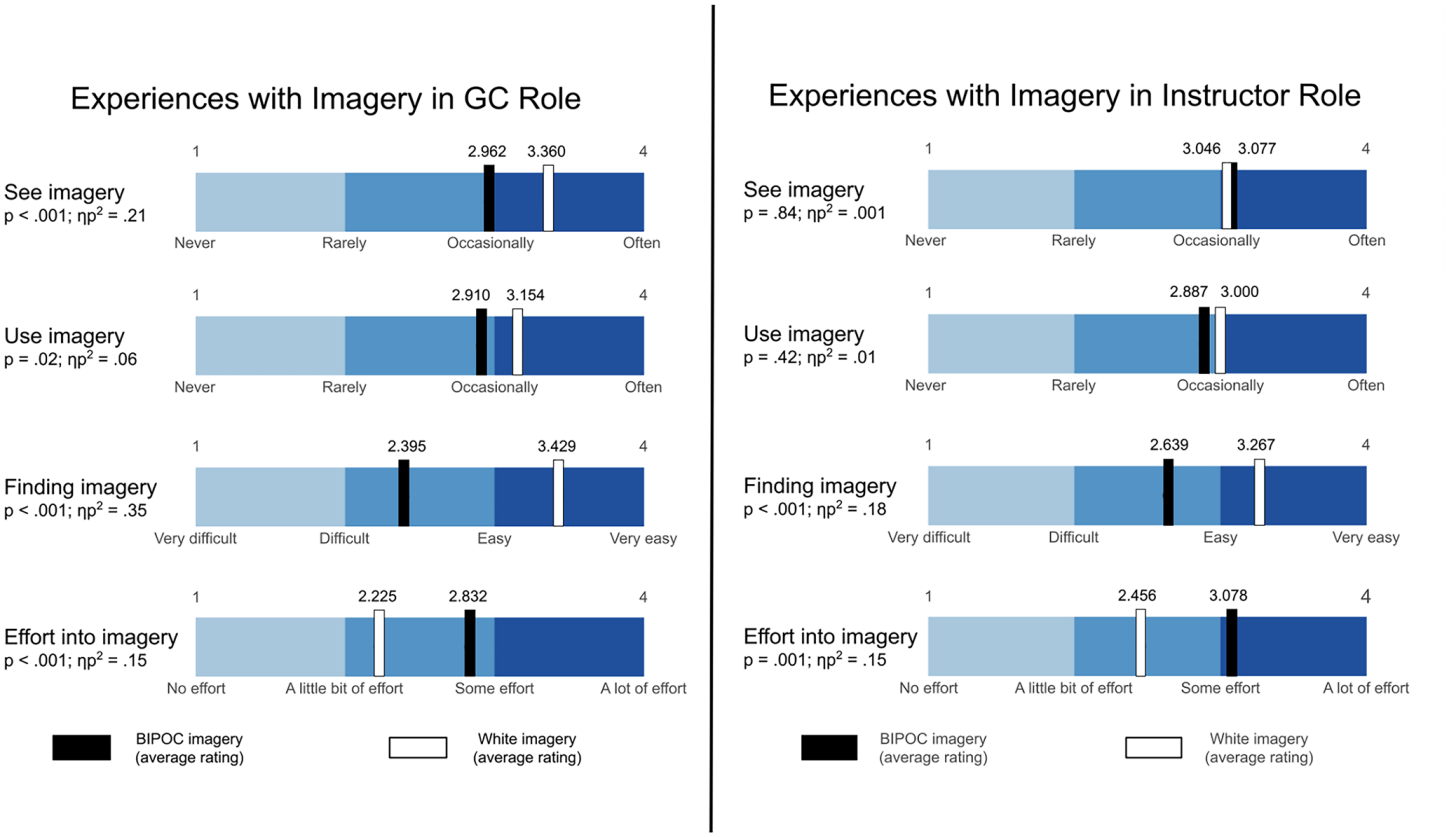
Experiences with imagery in GC role and instructor role.

We conducted a 2 × 2 mixed MANOVA to assess differences in responses between white (*n* = 70) and BIPOC (*n* = 18) GCs on their experiences with white and BIPOC imagery (i.e., frequency in seeing them, frequency in using them, difficulty finding them, amount of effort put into finding them). Though there was no significant difference in how white or BIPOC participants responded to these criteria (Wilks's λ = 0.978; *F*(4,83) = 0.47; *p* = 0.76), there was a notable significant difference in participants' overall experiences with white imagery compared to BIPOC imagery (Wilks's λ = 0.643; *F*(4,83) = 11.53; *p* < 0.001; $\eta_{p}^{2}$ = 0.36). As shown in Figure 1, participants saw (*p* < 0.001) and used (*p* = 0.02) white imagery more frequently than BIPOC imagery. Furthermore, they had a more difficult time finding BIPOC imagery (*p* < 0.001) as well as put more effort into looking for BIPOC imagery (*p* < 0.001).

### 3.3 Genetic counseling program instructors' experiences with medical imagery

We conducted a 2 × 2 mixed MANOVA to assess differences in responses between white (*n* = 53) and BIPOC (*n* = 13) instructors on their experiences with white and BIPOC imagery using the four aforementioned criteria. There was no significant difference in how white versus BIPOC instructors responded (Wilks's λ = 0.985; *F*(4,61) = 0.23; *p* = 0.92; $\eta_{p}^{2}$ = 0.02), but there was a difference in participants' overall experiences with white and BIPOC imagery (Wilks's λ = 0.698; *F*(4,61) = 6.61; *p* < 0.001; $\eta_{p}^{2}$ = 0.30). Participants had a harder time finding BIPOC imagery (*p* < 0.001), and they put more effort into finding BIPOC imagery (*p* = 0.001). There was no significant difference in how often they saw (*p* = 0.84) or used (*p* = 0.42) BIPOC imagery compared to white imagery (Figure 1). Post‐hoc paired *t*‐tests of frequency of using BIPOC imagery based on the type of class taught (e.g., counseling skills, medical genetics, ethics, research) found no differences (*p* range: 0.06–0.84). There was a significant correlation (*r*(53) = 0.32, *p* = 0.02) such that the more likely participants were to explain physical differences between white and BIPOC patients, the more they used BIPOC imagery.

Of those who reported having both a GC role and an instructor role (*n* = 64), a repeated measures MANOVA found no significant difference in participants' experiences with white and BIPOC imagery between their two roles (*p* = 0.07), though the effect size was just below the cutoff for large ($\eta_{p}^{2}$ = 0.13). Using BIPOC imagery in one role was positively correlated with use in the other (*r*(74) = 0.62, *p* < 0.001), meaning use tended to be consistent across roles.

### 3.4 White and BIPOC imagery in genetic counseling curriculum

Participants who held an instructor role (*n* = 66) noted they were more likely to use images of white to BIPOC bodies (*p* < 0.001; *d* = 0.81) in their lecture materials. They did not report significant differences in how often white versus BIPOC images were present in textbooks (*p* = 0.19), articles (*p* = 0.23), or videos (*p* = 0.29) provided to their students. Of note, several instructors were unsure of how often white and BIPOC imagery was used in these materials; they were excluded from this analysis. Instructors reported they occasionally provided explanations on the physical differences between white and BIPOC patients (*M* = 2.92; 1 = never, 4 = often). When applicable to their lesson or class, they felt somewhat confident in explaining these differences (*M* = 3.45; 1 = very confident, 5 = not at all confident).

Ninety‐two participants reported attending and graduating from a genetic counseling program. When participants reflected on their student experience, they reported that they were significantly more likely to have seen white images than BIPOC images in their curriculum (*p* < 0.001). Furthermore, they rarely received explanations of the physical differences between white and BIPOC patients (*M* = 2.24). After graduation, participants felt slightly unprepared to recognize genetic conditions in patients regardless of their race (*M* = 2.79; 1 = strongly agree [in preparedness], 4 = strongly disagree).

### 3.5 Access to diverse imagery

**FIGURE 2:**
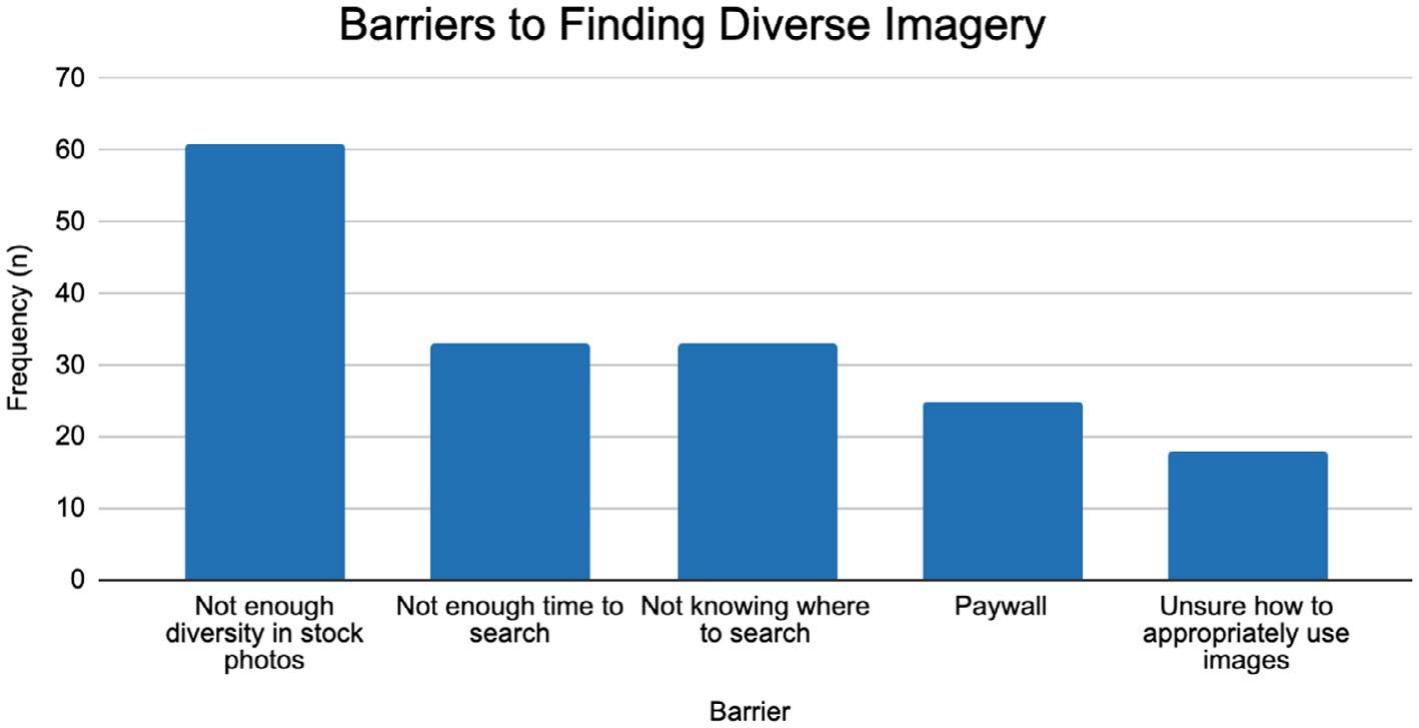
Barriers to finding diverse imagery. Participants were able to select multiple barriers they have experienced.

All participants, regardless of their roles, were queried about their imagery use. These participants reported they were more likely to use diverse imagery if there were an easy way to access them (*M* = 3.46, 1 = strongly disagree, 4 = strongly agree), and the most common barrier they experienced is the lack of diversity within stock photography resources (*n* = 64; Figure 2). When asked to rank their preferences on resources to find diverse imagery, the three most popular were stock photography, published literature, and research websites (e.g., GeneReviews; Table 2).

**TABLE 2:** Ranked list of preferred resources to obtain diverse imagery.

Type of resource	Average rank
Stock photography	3.16
Published literature	3.47
Research websites	3.76
Textbooks	3.82
Shared repository	4.63
Pamphlets	5.28
Word of mouth	5.98
Grants to hire artists	6.18

*Note*: 1 = most preferred.

The most common places from which they obtain/would obtain diverse imagery include images from a search engine (*n* = 44) and textbooks (*n* = 18). When searching, participants mainly use/would use terms that encompass both the condition and the race/skin tone/ethnicity of interest (*n* = 16). Examples of specific resources and search terms mentioned by the participants can be found in Table 3.

**TABLE 3:** Summary of resources and search terms used by participants.

Category	*n*	Examples stated by participants
Resources		
Images from a search engine	44	“Google”, “Google Images”, “Bing Images”
Books/textbooks	18	“Thompson & Thompson text”, “atlas of human malformation”, “genetics textbook”
Advocacy/patient websites	6	“rare disease organization websites”, “disorder‐specific support websites”, “cancer.gov PDQ images”, “patient advocacy groups specifically for people of color”
Published literature and research websites	5	“PubMed”, “GeneReviews”, “case reports”
Stock photography/image bank on the Internet	5	“Greenwood Genetics counseling aids”, “Canva”, “nappy.co”, “open source images”
Image bank from job	4	“physician log of images”, “patient photos [from institution]”, “paid resources from health orgs”
Social media	4	“Tik Tok”, “Instagram account @ebereillustrate”, “social media”
Other	5	“videos”, “old slide decks from labs”, “hired an artist to create images”
Search terms		
Race/skin/ethnicity + condition/medical	16	“the name of the condition and the patient's race”, “non‐white patient with [condition]”, broad search of the condition then later specifying race
No specific search terms	7	Scrolling until something is found
Condition/medical‐related terms only	5	“pathology”, “same terms for non‐diverse images”, “name of condition/medical feature”
Race/skin tone/ethnicity‐related terms only	4	“Non‐White”, “people of color”, specific race/ethnicity
Other	10	“Genetics in the United States”, “images of human bodies”

Overall, participants reported that an increase in diversity of the images shown in their curriculum would have positively impacted their genetic counseling training (*M* = 3.46; 1 = strongly disagree, 4 = strongly agree). Similarly, increased diversity of images would positively impact their preparatory duties (*M* = 3.39).

## 4 DISCUSSION

Though the composition of diverse imagery has been explored in some medical specialties (e.g., dermatology) where skin color directly impacts symptom assessment, this is one of the first known studies to investigate its use within the genetic counseling role and the genetic counseling program curriculum. Genetic counselors do not see or use BIPOC imagery as often as white imagery, though this difference was not as statistically significant among instructors. Both groups, however, reported having a more difficult time finding BIPOC imagery and have had to put more effort into finding it. Similarly, it was uncommon for participants to see BIPOC images or receive explanations about the phenotypical differences between BIPOC and white patients during their time as students. These results highlight the need for increased diversity in image resources so as not to exacerbate the health disparities among patients of color.

### 4.1 Societal influences on medical imagery

When the participants reflected upon the time they were students in their GC program, they reported that they were more likely to see white imagery compared to BIPOC imagery and that their instructors rarely explained how genetic conditions can present differently in white and BIPOC patients. Thus, they felt somewhat unprepared to recognize these differences after graduation. On the other hand, there was no significant difference in the overall use of white and BIPOC imagery for instructors, though they did use white images more frequently in their lectures. Because genetic counseling training programs often utilize textbooks and resources from other medical specialties due to the scarcity of GC‐specific texts, this may contribute to the predominance of white imagery in lecture materials. Future studies could explore why lectures in particular are more likely to have white imagery while other educational resources have similar amounts of white and BIPOC imagery. Increasing diversity in medical imagery across healthcare would support a more collaborative educational environment and help close gaps in training and research.

Instructors reported explaining phenotypic differences between patients of varying races more often than participants' reflections as students noted. This discrepancy of experiences between past students who have graduated and current instructors is likely due to time. The instructors were asked about their experiences with medical imagery during the 2022–2023 academic year, whereas the mean and median graduation years within our sample were 2016 and 2018, respectively. Further research is needed to clarify whether this discrepancy reflects differences in recall, self‐reporting, or actual changes in practice over time.

There has been an increased emphasis on diversity, equity, and inclusion (DEI), most notably in light of the murder of George Floyd and the subsequent Black Lives Matter movement in 2020. Some GC programs have made efforts to fund, support, and recruit more applicants from underrepresented communities, including a consortium formed in 2021 by five GC programs called the Alliance for Genetic Counseling Fellowship (Kotz, 2022). The field did experience a subtle increase in diversity. From 2019 to 2023, the percentage of Black GCs increased from 1% to 2%, Asian GCs from 8% to 9%, and Hispanic/Latine GCs from 2% to 3%, though American Indian/Alaska Native and Native Hawaiian/Pacific Islander GCs each remain less than 1% of the field (NSGC, 2019). The initiatives conducted to diversify the medical field are one example of the importance of amplifying the voices and experiences of BIPOC communities.

Instructors were more likely to use images of white bodies in their lecture materials. Interestingly, they reported no significant differences in how often white versus BIPOC imagery was present in textbooks, articles, and videos. This may be partially attributed to the uncertainty among several participants regarding how often white and BIPOC bodies appeared in these resources, which is further reflected by the widespread preference of both GCs and instructors to search for imagery with online search engines. Though participants who hold both roles likely find imagery in similar places, that imagery may be used differently across roles. GCs were more likely to encounter white imagery than BIPOC imagery compared to instructors, despite there being no significant difference in participants' overall experiences with white and BIPOC imagery between the two roles. This discrepancy may reflect how clinical GCs are potentially limited to preexisting resources that are tailored to a particular medical specialty. In contrast, instructors might have more freedom in selecting images for lectures.

Participants expressed a willingness to use diverse imagery if readily available, but they indicated numerous challenges in accessing it. The most frequently reported barrier was a general lack of diversity in stock photo resources. This barrier is one of many that have resulted in BIPOC imagery being more arduous to find. While diverse imagery was acknowledged as beneficial in educational and professional settings, the reliance on readily available resources, such as Google searches, stresses the need for increased diversity in mainstream repositories. Moreover, the practice of pairing desired conditions with specific racial or ethnic identities to find relevant imagery highlights the prevalence of a white default in visual representations, necessitating a broader need for inclusivity in image creation. Aside from Google, participants also want to find diverse medical imagery from academic resources such as published literature, research websites, and textbooks. The underrepresentation of BIPOC in research likely contributes to this (Committee on Improving the Representation of Women and Underrepresented Minorities in Clinical Trials and Research, 2022; Florentine et al., 2022; National Institutes of Health, 2021). Thus, it is important to recruit more BIPOC in education and research efforts.

### 4.2 Study limitations

One limitation of this study was that we did not reach our target sample size and had a low response rate of 3.4%. We were able to detect effects of at least moderate size with a power of 0.80 per our sensitivity analysis, so the likelihood of practically meaningful Type II errors is small. The beginning of the survey included a CAPTCHA prompt, but it is likely that this was not a foolproof method of eliminating bots. For example, the fill‐in‐the‐blank questions had a low response rate and were prone to duplicate and illogical responses. Though we excluded bot responses to the best of our ability, it is possible that some were included in the final analysis.

Participants also self‐reported their use of medical imagery. This, in conjunction with potential ascertainment and social desirability biases, could have skewed this sample's perception of imagery and diversity, thus lessening the generalizability of this study's results to the genetic counseling field. Therefore, the repetition of this study with a larger sample is encouraged.

Other limitations lie within the survey design. Participants were asked how much effort they put into looking for BIPOC and white imagery, but the reason for the effort is unclear. Do participants put more effort into finding BIPOC imagery because of its scarcity or because they personally want to find it? Likewise, participants were asked how often they use imagery. This question did not consider the difference in using imagery *if* readily available compared to if it is *not* readily available, nor did it factor in the overall use of visual aids. Participants may have stated they rarely use BIPOC imagery because of its scarcity or because they rarely use visual aids in general. We were unable to find a published estimate of the frequency of visual aid use by genetic counselors in either clinical practice or education, so we were unable to assess how representative our sample is in terms of imagery use. This potential issue may also affect some course types more than others (e.g., medical genetics vs. research methods). Errors in the phrasing of some survey questions may have resulted in varying interpretations and thus affect the statistical validity of their experiences.

### 4.3 Research directions

Given that we could find no previous studies addressing diverse imagery in genetic counseling, this study was exploratory in nature. One area of further study is how genetic counseling students today interact with diverse medical imagery. This may offer a perspective that more closely aligns with that of the instructors and better assesses the landscape of imagery and genetic counseling curriculum nowadays. The Accreditation Council for Genetic Counseling (ACGC) outlines the standards for genetic counseling programs as well as guidelines for the training of genetic counselors to ensure their success in the field. The Standards for Accreditation state that programs must include training on, but not limited to, implicit biases, personal identities, discrimination, and how patients of color interact with the healthcare system (Accreditation Council for Genetic Counseling, 2023b). Similarly, one practice‐based competency for genetic counselors is the ability to “demonstrate how disparities, inequities, and systemic bias affect access to healthcare for diverse populations” (Accreditation Council for Genetic Counseling, 2023a). Given these standards, it is hypothesized that future studies on genetic counseling curricula would suggest greater diversity in medical imagery compared to this study. Whereas the standards for diversity, equity, and inclusion training are clear for genetic counseling programs, those of clinical settings may vary greatly from site to site. Therefore, it is important to continue the development of resources that genetic counselors, instructors, and medical providers can easily access.

Participants who were both a GC and an instructor did not have significantly different experiences with medical imagery between their two roles. With a moderate‐to‐large effect size of 0.13, however, this does suggest further exploration should be undertaken. These participants may look for imagery in the same places for both roles, but given a sample of 64 who indicated both roles, this may warrant further investigation. We did not ask about years of overall experience as a genetic counselor or instructor in our study. Exploring the relationship between years in the field and imagery use could provide valuable insights, as GCs and instructors may rely on the same resources throughout their careers, potentially contributing to the lack of diversity in imagery.

This study focused on race, ethnicity, and skin tone in medical imagery. However, there are many other facets of diversity. More research needs to be done in evaluating the diversity of sex, gender, religion, age, and disability, as well as how these identities intersect in medical imagery and condition descriptions. Furthermore, most instructors were unsure of how often BIPOC imagery appears in educational materials provided to their students. Studies have been done to assess images within medical textbooks (Louie & Wilkes, 2018), but this has yet to be done in genetic counseling education textbooks and other materials.

### 4.4 Practice implications

For genetic counselors to effectively serve diverse communities, it is imperative to expand education on the experiences and challenges faced by BIPOC individuals. While genetic counselors do not clinically diagnose genetic conditions, understanding how these conditions manifest across different patient demographics is essential. This knowledge validates patient experiences, streamlines the diagnosis process, and enables sessions and resources to be tailored to meet individual needs. Additionally, incorporating diverse imagery can help mitigate biases surrounding genetic conditions among both healthcare providers and patients. For instance, cystic fibrosis, while affecting individuals of all races and ethnicities, is often perceived as a condition that is exclusive to those of European ancestry (Wu et al., 2024). Newborn screening and genetic testing panels tend to exclude rare variants that are more likely to be present in BIPOC individuals. Misconceptions that lead to the underdiagnosis of patients of other ethnicities accentuate the importance of diverse representation in medical imagery to dispel such biases. Moreover, diverse imagery can empower patients by fostering inclusivity and challenging the perpetuation of a white‐centric standard within healthcare practices. Given that current ACGC curriculum requirements do not specifically address medical imagery, ACGC could consider requiring the inclusion of diverse imagery in genetic counseling training.

The objective of enhancing diversity in medical imagery is likely to be achieved through collaborative efforts, as diverse imagery can strengthen all healthcare disciplines. Genetic counselors could form coalitions with medical geneticists to increase awareness of these differences within the field. Additionally, the medical community could work with medical illustrators to create a repository of diverse images that feature a range of skin tones. However, some participants have identified a paywall as a barrier to accessing diverse images. To address this issue, the National Society of Genetic Counselors (NSGC) could consider purchasing the rights to these images, thereby making them available to NSGC members.

## AUTHOR CONTRIBUTIONS

L. Sanders helped to conceptualize the study, interpreted the data, wrote the manuscript, helped to edit and evaluate the manuscript, acted as the corresponding author, and was responsible for data access and integrity. K.M. Davalos and I. Kashmola‐Perez helped to edit and evaluate the manuscript. I.M. MacFarlane performed statistical analysis, interpreted the data, helped to edit and evaluate the manuscript, and was responsible for data access and integrity. H. Lee helped to conceptualize the study, supervised the development of work, interpreted data, helped to write, edit, and evaluate the manuscript, and was responsible for data access and integrity. All authors have given final approval of the version to be published.

## CONFLICT OF INTEREST STATEMENT

The authors have no conflicts of interest to declare.

## DISCLOSURE

AI statement: No artificial intelligence tools were used to develop this manuscript.

## ETHICS STATEMENT

This study received approval from the University of Minnesota Institutional Review Board (STUDY00020057). Informed consent was obtained from all participants before the study. No nonhuman animal studies were carried out by the authors for this article.

## Data Availability

The data that support the findings of this study are available from the corresponding author upon reasonable request.
